# Substitution of gp120 C4 region compensates for V3 loss-of-fitness mutations in HIV-1 CRF01_AE co-receptor switching

**DOI:** 10.1080/22221751.2023.2169196

**Published:** 2023-02-28

**Authors:** Yueyang Yu, Yi Feng, Zehua Zhou, Kang Li, Xiaoyan Hu, Lingjie Liao, Hui Xing, Yimig Shao

**Affiliations:** a School of Medicine, Nankai University, Tianjin, People’s Republic of China; b State Key Laboratory for Infectious Disease Prevention and Control, National Center for AIDS/STD Control and Prevention, Chinese Center for Disease Control and Prevention, Beijing, People’s Republic of China; c Changping Laboratory, Beijing, People’s Republic of China

**Keywords:** HIV-1, tropism, coreceptor-switching, C4 region, V3 loop

## Abstract

HIV-1 infection is mediated by a viral envelope subsequently binding to CD4 receptor and two main coreceptors, CCR5 (R5) for primary infection and CXCR4 (X4) in chronic infection. Switching from R5 to X4 tropism in HIV-1 infection is associated with increased viral pathogenesis and disease progression. The coreceptor switching is mainly due to variations in the V3 loop, while the mechanism needs to be further elucidated. We systematically studied the determinant for HIV-1 coreceptor switching by substitution of the genes from one R5 and one X4 pseudoviruses. The study results in successfully constructing two panels of chimeric viruses of R5 to X4 forward and X4 to R5 reverse switching. The determinants for tropism switching are the combined substitution of the V3 loop and C4 region of the HIV-1 envelope. The possible mechanism of the tropism switching includes two components, the V3 loop to enable the viral envelope binding to the newly switched coreceptor and the C4 region, to compensate for the loss of fitness caused by deleterious V3 loop mutations to maintain the overall viral viability. The combined C4 and V3 substitution showed at least an eightfold increase in replication activity compared with the pseudovirus with only V3 loop substitution. The site-directed mutations of N425R and S440-I442 with charged amino acids could especially increase viral activity. This study could facilitate HIV-1 phenotype surveillance and select right entry inhibitor, CCR5 or CXCR4 antagonists, for antiviral therapy.

## Introduction

The entry of HIV-1 into the target cells is through the subsequent binding of its envelope protein to the CD4 receptor and chemokine coreceptors on the cells [1,2]. It has been shown that the coreceptors binding is mainly between the third variable region (V3 loop) of the viral envelope [3,4] and the second extracellular loop (ECL2) and the amino-terminal domain (NT) of the Chemokine receptor [5]. There are two main types of coreceptors used by HIV-1, CCR5 (R5) in most primary infection and CXCR4 (X4) in chronic infection [5,6]. HIV-1 binds to the two types coreceptors called R5 or X4 tropism strains respectively. The transition from R5 to X4 tropism during HIV-1 infection is called coreceptor tropism switching or R5 to X4 switching [7,8]. Past studies exploring the determinants of viral tropism and coreceptor switching mainly focused on the V3 loop [2,9] and less on the other viral variable and constant regions (C regions) [10,11]. Some mutations introduced to the V3 region could alter coreceptor utilization or even lead to coreceptor switching, but many of the variants have a greatly reduced viability. This poses a genetic barrier for survival fitness to the coreceptor switching, which can be compensated by the structure of other V or C regions [12,13]. A slight conformational change in the C4 region can influence the structure of V3 [14–16], and sequence variants within the C4 domain are also involved in coreceptor selection. The C4 region and V3 have structural interactions and functional impacts on viral recognition, CD4 binding, and virus tropisms [17–20].

Different HIV-1 subtypes and circulating recombinant forms (CRF) have different tendencies or prevalence of R5 or X4 tropism viruses. The CRF01_AE is one of the HIV-1 subtypes that has spread widely in Southeast Asia and China with characteristics of higher X4 viruses and rapid disease progression [21,22]. Our previous study found that CRF01_AE cluster 4 virus-infected patients are associated higher prevalence of X4 viruses and lower CD4 counts, compared to that of CRF01_AE cluster 5 infected people [23]. This shows there are constant viral genetic and phenotypic evolutions in HIV-1 subtypes and clusters, associated with R5 to X4 switching. The studies have shown that R5 tropism viruses can be isolated at all stages of HIV-1 infection, while the R5/X4 dual and X4 only tropism strains generally emerged at later stage infection, marked by a decline of the immune defences [24,25].

With the introduction of entry inhibitors into the clinic as a component of antiretroviral treatments, there has heightened the interest in coreceptor utilization and switching conditions of HIV. The HIV-1 coreceptor switch from R5 to X4 associated with poorer clinical prognosis has been observed in many patients on antiviral therapy and can be assumed to be one pathway leading to resistance of CCR5 inhibitors in clinical trials [6]. Therefore, exploring and analysing molecular properties of the virus associated with the R5 to X4 switch contribute to medication guidance and disease control. Based upon the previous research and the virus isolated from the samples of men-who-have-sex-with-men (CYM cohort) [23], our research started by illustrating the virus strains of different coreceptor utilization and their biological basis of switching, with the expectation of providing some references to the mechanism of virus-coreceptor interaction and HIV antiviral therapy.

## Materials and methods

HIV-1 plasma samples of patients 167, 248, and 194, from whom viruses with CCR5 tropism, CXCR4 tropism, and dual-tropism were isolated respectively were used from the Beijing Chaoyang MSM cohort (the CYM cohort) for this study [23]. Viral isolate 176 was also derived from this cohort. RNA was extracted from plasma or virus samples and subsequently reverse transcribed to cDNA. Single genome amplification (SGA) of the cDNA was performed to obtain the *rev/env* cassette by nested PCR for two-round amplification. PCR amplification was carried out with PrimeSTAR® Max DNA Polymerase (TaKaRa Bio, JP). The nested PCR primers were as follows: first round sense primer HZAEOB 5′-TAGAGCCCTGGAATCATCCGGGAAG-3′, first round antisense primer HZAEOE 5′-TTACTACTTGTTACTGCTCCATGT-3′, second round sense primer HZAEIB 5′-CACCTTAGGCATCTCCTATGGCAGGAAGAAG-3′ and second round antisense primer HZAEIE 5′-ATCTAGATCTTGAGATACTGCTCCTAC-3′.

The PCR products were purified and cloned into the pcDNA 3.1D/V5-His-TOPO (Invitrogen, Carlsbad, CA, USA) according to the instructions. These three plasmids, p167, p248, and p194, make sequencing validation for the subsequent use of reconstruction and mutagenesis.

Several parts of the env gene, mainly V3 and C4, were substituted or chimeric in the template plasmids p167, p248, p194, and the isolated virus 176. We introduced 15–25 bp homologous sequence of the vector into both ends of the target fragment. After linearization of the plasmid, it was ligated to the target fragment by a seamless cloning kit (Transgene Biotech, Beijing, China) according to the instruction. We performed site-directed mutagenesis by PCR amplification using a pair of primers by extending 15 bp toward both ends centred on the nucleotide requiring mutation. The products obtained above were subjected to transform into competent cells to obtain plasmids capable of producing pseudoviruses.

Plasmids above were co-transfected with env-deficient HIV-1 backbone pSG3Δenv into 293 T cells at a ratio of 1:2. Forty-eight hours after transfection, supernatants were harvested and titrated in TZM-bl cells and chemokine receptor antagonists are also added to identify the utilization of coreceptor. Ghost-CD4-CCR5 and Ghost-CD4-CXCR4 cell lines were quantitatively infected with psuedovirus supernatant of 1000TCID_50_. If the viral titre did not reach the requirement for dilution, the maximum volume of 500 μl shall be used for infection. After incubation for 48 h, the cells were examined under a fluorescence microscope or flow cytometer. Tissue culture infective dose (TCID) level and half inhibiting concentration (IC_50_) were calculated using the Reed-Muench method. Data were plotted using GraphPad Prism 8.

The structure of gp120 sequence of 167-248V3 (C1) and 167-248V3-S440D-I442R (M9) is obtained by homologous modelling in the Robetta program (https://robetta.bakerlab.org/). The structural model of Chemokine receptor CXCR4 derived from the Protein Database (PDB: 4rws). We used PyMOL molecular graphics system to align the structures of gp120 and coreceptor described above into the cryo-electron microscopy structure (PDB: 6meo). The mutagenesis module of PyMOL was used to mutate amino acids of 425 and 440–442 in the C4 region, respectively. Chemical bond and electrostatic analysis were performed using features that are included in the software.

## Results

### Construction and tropism identification of backbone plasmids

**Figure 1.**
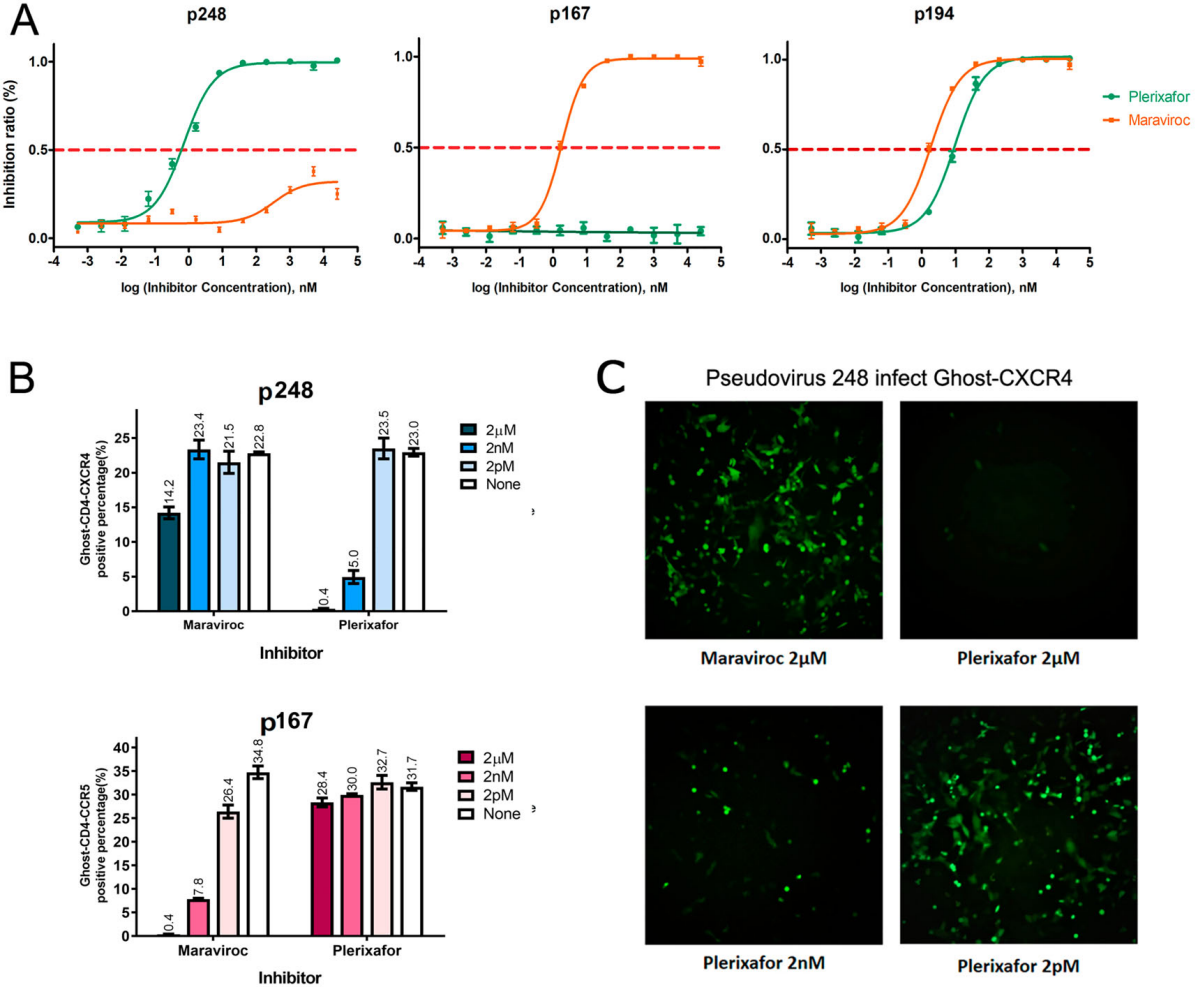
Determination of cell tropism for the pseudovirus 167 and 248. (A) IC_50_ of pseudovirus 248, 167, and 194 in a single-cycle infection assay was displayed in TZM-bl cells. The pseudovirus 248 is strongly inhibited by CXCR4 inhibitor Plerixafor (IC_50_ of 0.69 nM) and weakly inhibited by CCR5 inhibitor Maraviroc. The pseudovirus 167 is strongly inhibited by CCR5 inhibitor Maraviroc (IC_50_ 1.8 nM) and cannot be inhibited by Plerixafor. Pseudovirus 194 could be inhibited by Maraviroc and Plerixafor with the IC_50_ of 1.85 nM and 9.68 nM respectively. (B) The inhibitory effect in Ghost-CD4 cell lines of two coreceptor antagonists on p167 and p248 at concentrations of 2 pm, 2 nm and 2 μm, respectively. (C) The pseudovirus p248 induced obvious GFP expression in the Ghost-CD4-CXCR4 cell line. The background level of fluorescence is weakly blocked by the CCR5 inhibitor Maraviroc (2 µM) but can be almost completely blocked by 2 µM of CXCR4 inhibitor Plerixafor, and the effect of Plerixafor-mediated inhibition was dose dependent.

Since the HIV-1 envelope is responsible for coreceptors binding, we first constructed envelope gene expression plasmids p167 and p248 from the plasma sample of patients 167 and 248 from the CYM cohort. The identification of viral tropism in vitro needs to be determined not only by Ghost cell lines binding assay but also by a specific receptor antagonist inhibition assay, since a low level of CXCR4 could be endogenously expressed in the Ghost-CD4-CCR5 cell line, resulting in an X4-tropic virus that can bind both a large number of Ghost-CD4-CXCR4 and a small number of Ghost-CD4-CCR5 cells. Plerixafor (AMD3100) is a small molecule inhibitor of CXCR4, which suppresses infection of HIV selectivity toward X4-tropic virus rather than dual-tropic. When using Ghost-CD4 cell lines to identify the coreceptor utilization of these two template pseudoviruses, we found that 167 only infected Ghost-CD4-CCR5, while 248 could infect both Ghost-CD4-CCR5 and Ghost-CD4-CXCR4 in the percentage of 39% and 9.7% respectively. But the inhibition assay of chemokine antagonist shows different results. Plerixafor suppresses infection by p248 with an IC_50_ of 0.69 nM performed on TZM-BL, while the IC_50_ of Maraviroc inhibiting p248 is far more than 100 μM (Figure 1A). It can be seen from Figure 1(B and C) that the ability of pseudovirus p248 to infect Ghost-CD4-CCR5 cells could not be inhibited by high concentration of Maraviroc (CCR5 antagonist), but the ability to bind Ghost-CD4-CXCR4 cells could be inhibited by low dose of CXCR4 receptor antagonist Plerixafor in a dose-dependent manner. This result indicates that although pseudovirus 248 can infect both Ghost-CD4-CCR5 and Ghost-CD4-CXCR4, it shows X4-tropism, because of a low level of CXCR4 endogenously expressed in Ghost-CD4-CCR5 [26].

### Infective activity and coreceptor binding of the chimeric pseudovirus

**Figure 2.**
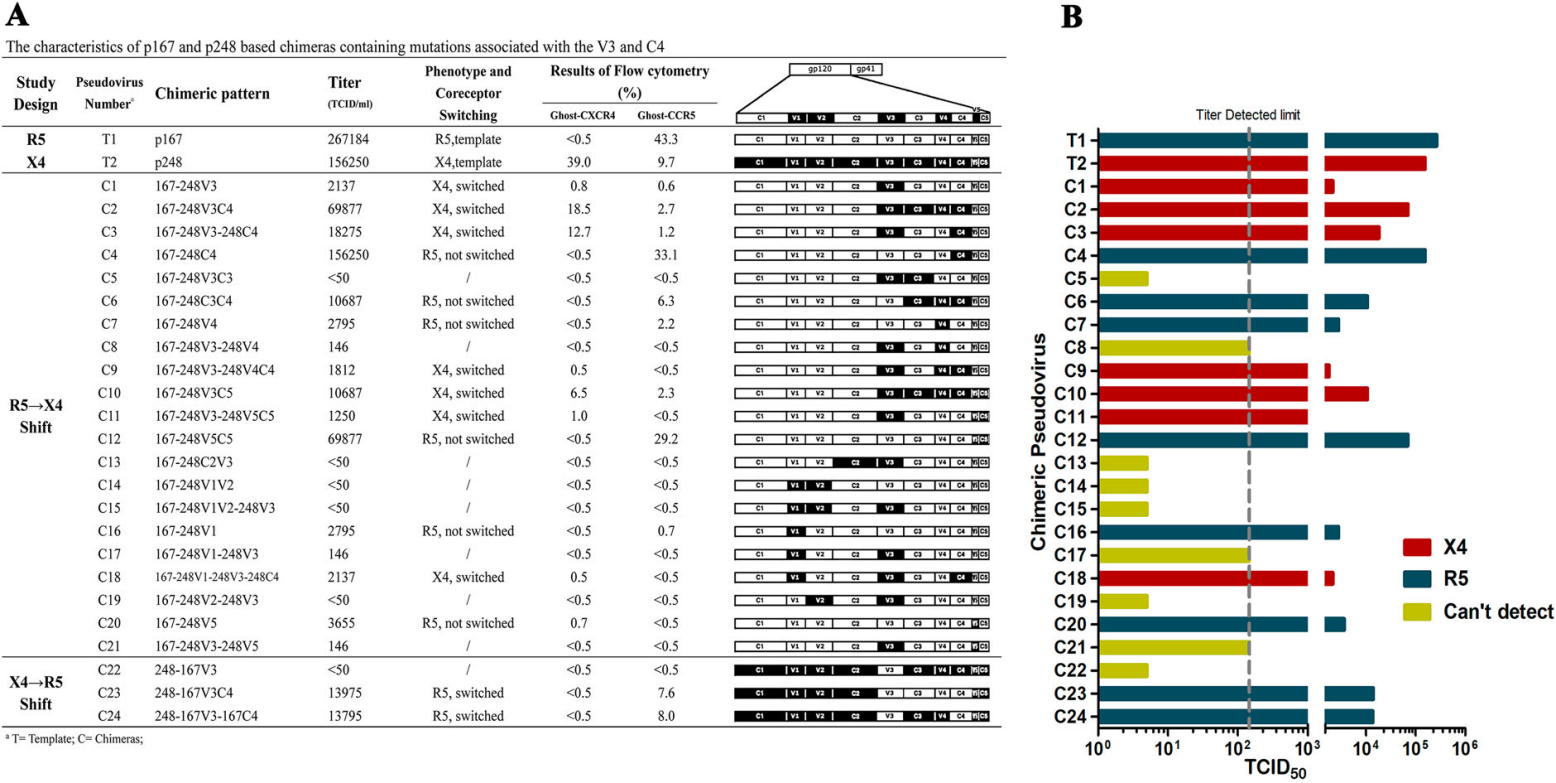
Entry efficiency and tropism of chimeric pseudovirus mutants in a single-cycle infection assay employing TZM-bl cell and Ghost-CD4-CCR5/Ghost-CD4-CXCR4 cells. (A) TCID_50_ and the percentage of fluorescent positive cells are further detailed. (B) Pseudoviruses that mediate entry via CCR5 are shown in blue, pseudoviruses that mediate entry via both CCR5 and CXCR4 but more efficiently via CXCR4 are shown in red, and pseudoviruses that can’t detect fluorescence by either Ghost-CD4-CCR5 or Ghost-CD4-CXCR4 are shown in yellow.

To find the gene segments responsible for virus tropism switching from R5 to X4 or R5/X4-dual tropisms, we systematically substituted the gene segments of gp120 from p167 with the p248. The plasmids are named in the manner of backbone gene-substituted region, and each plasmid has a serial number. For instance, p167-248V3-248C4 numbered as C3 represents a plasmid with p167 as the backbone gene, and both the V3 loop and the C4 region are replaced with the corresponding gene segments of the p248 plasmid. Another manner of naming needs to be mentioned, in the chimera 167-248V3C4 numbered as C2, the replaced region of backbone p167 contains all sequences from V3 loop to C4 region, including V3, C3, V4, and C4. We indicated the positions of the fragment substitutions for each chimeric pseudovirus in Figure 2(A). The replication activity of the chimeric pseudoviruses changed greatly after the substitution, not all of the chimeric viruses were infectious. The results of pseudovirus replication activity and coreceptor utilization are shown in Figure 2. With the V3 loop substitutions as a precondition, 24 chimeric pseudoviruses were constructed by independent or combinatorial substitution on gp120. Eight of the chimeric viruses remain CXCR5-tropism, seven viruses exhibit X4-tropism, and nine viruses have no infectious activity. It can be seen from Figure 2(A) that some of the pseudoviruses maintain the infectious activity after single fragment substitution but did not switch their tropism such as C7. Pseudoviruses containing substitution in or with the V3 loop were able to switch the utilization of the coreceptor, although the infectious activity might undergo a significant reduction to or even under the critical level of detection. Compared to the template pseudovirus p167, the single-round infectivity assay result of chimeric pseudovirus containing the V3 loop of p248 decreased by 125-fold (TCID_50_ of T1/C1 is 267184/2137). The results of coreceptor-utilization identification with Ghost-CD4 cell lines showed that the percentage of Ghost-CD4-CXCR4 positive cells was 0.8%, which was almost reduced to the same extent as the cut-off value for positivity (0.5% cut-off). Meanwhile, we also reverse substitute the V3 loop of p248 to p167, and the chimeric pseudovirus obtained by 248-167V3 (C22) showed altered tropism but still reduced infective activity. In terms of these two CRF01_AE subtype viruses, the change of V3 loop is accompanied by a sharp decline in infectious activity.

Although the V3 loop is critical for the coreceptor switching, the reduced viability of the virus resulting from the mutations in the V3 loop brings great uncertainty and interference to the subsequent identifications. In the process of exploring the effects of segment combinational substitution, we found that C4 significantly compensated for V3 loss-of-fitness mutations, resulting in an increased vitality of the virus. We chose the regions either structurally close to the V3 loop or previously described in other research associating with tropism, such as V1V2, to substitute together with the V3 region. The pseudoviruses obtained from the above chimeric plasmids were identified by cell assay and presented in Figure 2. Excitingly, the pseudovirus numbered C3 (p167-248V3-248C4), simultaneous presence of the C4 region with V3 loop of p248 on the p167 framework enables not only a coreceptor-switching but also elevated vitality of its pseudovirus. This compensatory effect on the vitality of the tropism-shifting virus was reflected by all chimeric pseudoviruses containing the C4 region, such as C2 and C10. Combinational substitution of the V3 loop with C4 region (C3, 167-248V3-248C4) showed an eightfold increase in the value of TCID_50_ (18275/2137) and the substitution of the region from V3 to C4 (C2, 167-248V3C4) showed a 32-fold increase in the value of TCID_50_ (69877/2137), compared with 167-248V3 (numbered as C1). However, no significant increase in replication activity could be seen in combinatorial substitutions of the V3 loop with the C3 region and V4 loop (C5 and C8). The two regions located between V3 and C4. The above evidence indicates that the presence of the C4 region is the minimum necessary existence to compensate for the reduced replication activity conferred by V3 alterations.

### Compensatory infectivity analysis of key amino acids in C4

**Figure 3.**
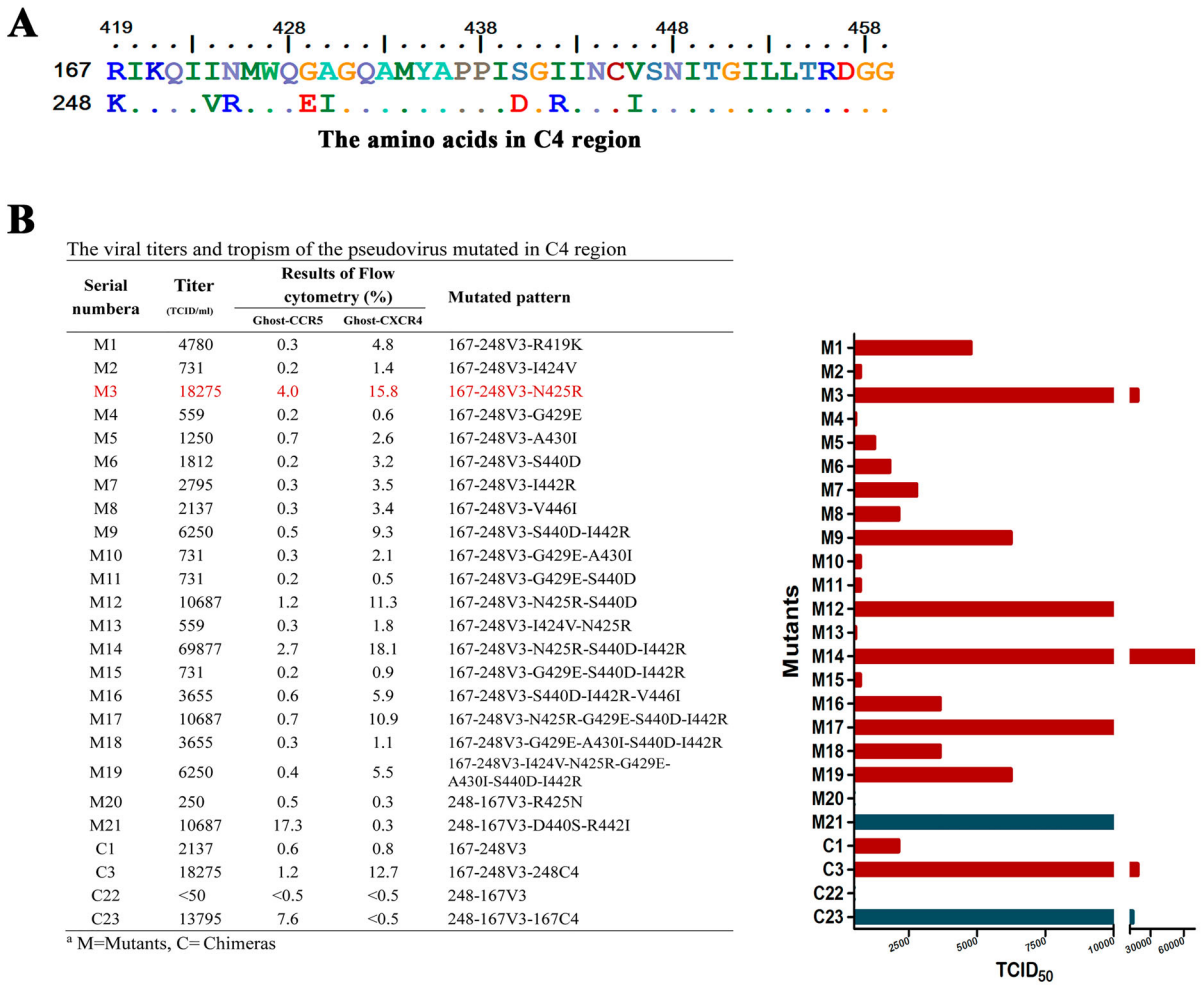
Eight different amino acids in C4 and its mutants. (A) The eight different amino acids in the C4 region of p167 and p248 are located at positions 419, 424, 425, 429, 430, 440, 442, 446, respectively (numbering based on HxB2). (B) Twenty-one mutants were designed with these eight amino acids and performed in p167-248V3 and p218-167V3, TCID_50_ and the percentage of fluorescent positive cells is further detailed. TCID_50_ is determined with TZM-bl cell. X4-tropic viruses are shown in red and R5-tropic viruses are shown in blue. Chimeras C1, C3, C22, and C23 are the control pseudoviruses. N425R showed the most significant enhancement of viral activity among the eight single-site mutations and highlighted in red colour in the table.

There are eight amino acid differences in the C4 region between p167 and p248 (Figure 3A). We made site-directed mutagenesis of these eight different amino acids in the C4 region on plasmid p167-248V3, one by one or in combinations, to test the role of each amino acid and to find the critical amino acids that could enhance viral replication activity. Because the results of cell assay in vitro are clear at a glance, we regard the changes in the positive proportion of Ghost-CD4 cell lines infected by mutated pseudoviruses as a measure of the binding capacity of viral antigens to co-receptors. Mutating these eight amino acids in the C4 region individually has little effect on overall replication activity improvement, except for N425R. Combinatorial mutagenesis and superimposed mutagenesis were subsequently performed, and pseudovirus vitality rose as the mutation in some key sites. The mutants and their vitality results are shown i*n* Figure 3(B).

Among the eight single-site mutations, N425R showed the most significant enhancement of viral activity. Mutation at this position alone was able to improve the overall replication activity of the virus by eightfold (18275/2137), which is close to the effect of whole C4 region replacement. The infectious activity of pseudoviruses containing N425R in multi-site mutation, such as M12, M14, and M17, has been significantly improved. Altering the charge properties of amino acids can often lead to greater changes in protein functions. Our combinatorial mutagenesis is based on these amino acids with different charge properties. Amino acids changed in positions 440–442 showed a unique increase in overall viral activity among the two-site mutations or multiple-site combination mutations. Compared with the other two-site mutations, the 440–442 mutant was more than twofold competent for increased replication activity. Mutations of S440D-I442R conferred significant enhancement of CXCR4 binding capacity. The introduction of this mutation in the chimeric pseudovirus C1 could obtain M9. Quantitatively infecting the two kinds of Ghost-CD4 cell lines with C1 and M9 shows that the binding capacity of M9 for Ghost-CD4-CCR5 does not change much, whereas the binding capacity of M9 for Ghost-CD4-CXCR4 greatly increased. It can also be seen in subsequent experiments that the introduction of this mutation makes the attenuation of the ability to infect Ghost-CD4-CCR5 while leaving the ability to infect Ghost-CD4-CXCR4 unchanged in p194 and 167-176V3 of the Table 1. To further confirm this result, we also conducted a reverse verification in pseudovirus 248-167V3 (C22). Compared with C22 before the introduction of reverse mutation D440S-R442I, M21 (248-167V3-D440S-R442I) showed a strong ability to infect Ghost-CD4-CCR5 cells (Figure 3B). The above results indicate that the mutation of S440D-I442R is an important site for the interaction between gp120 and CXCR4.

**Table 1.** The characteristics of p194 and Virus 176-based chimeras containing mutations associated with the V3 and C4.

Study design	Number^a^	Chimeric and mutated pattern	Titer (TCID/ml)	Coreceptor switching	Results of flow cytometry (%)	
					Ghost-CXCR4	Ghost-CCR5
**R5**	T1	p167	267184	R5, template	<0.5	43.3
**X4**	T2	p248	156250	X4, template	39.0	9.7
**R5/X4**	T3	p194	69877	R5/X4, template	9.9	13.5
**R5/X4**	V4^b^	Virus 176	/	R5/X4, template	/	/
**p194 associated**	C25	167-194V3	10687	R5/X4, switched	0.6	13.3
	C26	167-194V3-194C4	3655	R5/X4, switched	1.6	10.6
	M22	167-194V3-N425R	31250	R5/X4, switched	2.2	26.7
	M23	167-194V3-S440D-I442R	50	/	0.3	0.3
	M24	194-S440D-I442R	2137	R5/X4, switched	7.6	0.9
**Virus 176 associated**	C27	167-176V3	18275	R5/X4, switched	5.8	17.7
	C28	167-176V3-176C4	1250	R5/X4, switched	4.5	1.2
	M25	167-176V3-N425R	2795	R5/X4, switched	9.3	7.1
	M26	167-176V3-S440D-I442R	2137	R5/X4, switched	7.2	0.8

^a^ T = Template; C = Chimeras; V = Virus; M = Mutants.

^b^ The pseudovirus of CYM176 was not obtained, but the genes of its V3 and C4 were amplified for chimeric manipulation.

Viral isolates of CYM176 have been verified to be dual-tropic in the previous study.

**Figure 4.**
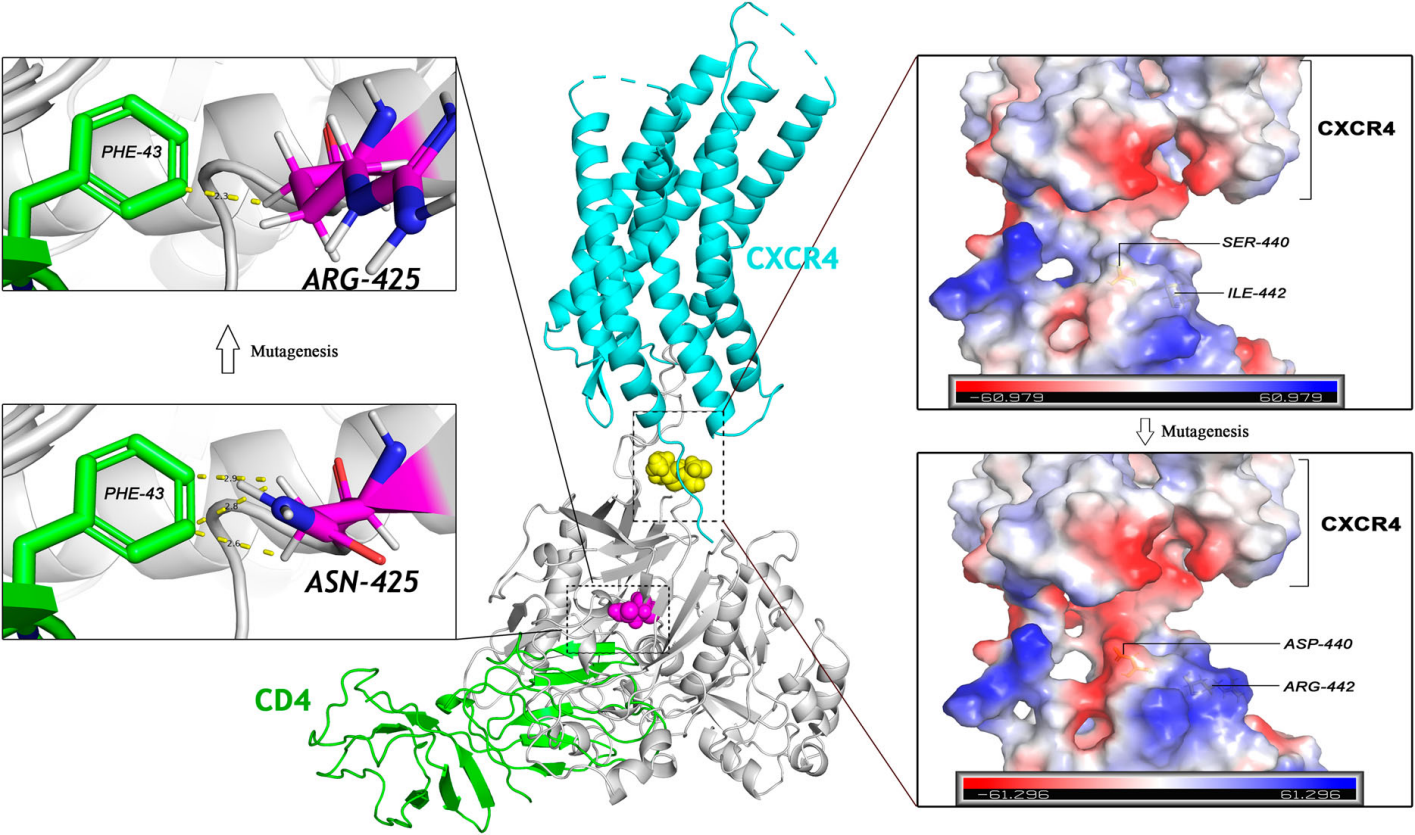
Structural modelling of chemokine receptor binding to key amino acid of C4. An overview of modelling complex of CD4-gp120-CXCR4 is in the middle of this figure. CD4 is shown in green, coreceptor shown in blue, and the gp120 of pseudovirus 167-248V3 (C1) is shown in grey. Critical C4 positions 425 and 440–442 coloured in magenta and yellow, respectively. The mutagenesis of N425R is indicated on the left. Three contacts with the distance of 2.6, 2.8, and 2.9 Å exist between the Asn425 and the Phe43 of CD4. Upon mutation of asparagine to arginine, the contacts between the two amino acids changed from 3 to a closer 2.3 Å one. The electrostatic potential energy of the model of pseudovirus C1 to CXCR4 is shown on the right side of the figure. The introduction of the mutation S440D creates a negatively charged pocket in the N-terminus of CXCR4. Positive charged amino acids are in blue and negative in red.

We preliminarily analysed how N425R and S440D-I442R mutations affect pseudovirus activity by using the cryo-electron microscopy structure (PDB: 6meo), which is the structural basis of coreceptor recognition by HIV-1 envelope spike. As shown in Figure 4, before mutation, asparagine, the 425th amino acid in the env of pseudovirus 167-248V3 (C1), had three bonds between chains within 3 Å contacted with Phe43 of CD4, with the distances of 2.6, 2.8, and 2.9 Å, respectively. After the mutation of amino acid 425 from asparagine to arginine, the interaction with Phe43 of CD4 changed to a single contact with a closer distance of 2.3Å. There are no chemical bonds less than 5 Å in distance between amino acids of CXCR4 and C4. But there are contacts between the V3 loop and C4 region in the same protein strand. The cryo-electron microscopy structure proved that the main contacts between gp120 and coreceptor N terminus are electrostatic and the X4 V3 loops generally have more positive charges than those of R5 viruses [27]. So we analyse the electrostatic of the mutated 440–442 amino acids. Before the mutagenesis of 440–442, there is almost no net charge interaction around S440. When position 440 becomes negatively charged aspartic acid, it forms a negatively charged pocket with the same negatively charged CXCR4 N-terminus, allowing the X4 V3 loop with positively charged, buried within it, to stably bind. The Ile442, located closer to the outer surface, mutates into positively charged arginine attracting the negatively charged amino acids in the N-terminus of CXCR4 to form a stronger binding force. The above modelling analysis explained to some extent the compensation mechanism of introduced mutations in the C4 region to the loss of replication activity caused by the change in the V3 loop.

### Use two more samples to verify the above results about mutated V3 and C4

It remains to be verified whether the above results and inferences about changes in tropism and viral infectivity derived from experiments on p167 and p248 can be applied to other HIV 01_AE subtype viruses. Two additional virus samples (CYM194 and CYM176) that had been confirmed to be dual-tropic in the previous study were selected for chimeric operation and mutagenesis. The results are shown in Table 1. Another pseudovirus clone p194 is a template plasmid constructed by the same method. It was identified as a dual-tropic pseudovirus by the inhibition test of the coreceptor antagonist Maraviroc and Plerixafor with the IC_50_ of 1.85 nM and 9.68 nM respectively (Figure 1A). We extracted RNA from viral isolate 176 and obtained the sequence of V3 by reverse transcription for fragments replacement. There was no major loss in overall infective activity upon substitution of the V3 loop of the dual-tropic viruses (p194 and Virus 176) on the p167 backbone. Therefore, even if the corresponding C4 region fragment was introduced into 167-194V3 and 167-176V3, it did not play a significant role in improving the viral vitality. It can be seen that V3 is indeed the most critical determinant of coreceptor utilization. Because after replacing the V3 loop of the dual-tropic strains, the backbone pseudoviruses that were originally R5-tropic possessed the ability to bind to both CXCR4 and CCR5 simultaneously (C25 and C27). Compared with the p194 (T3), the chimeric virus 167-194V3 (C25) seems to have different binding ratios to the two co-receptors. Ghost cell assay showed that C25, compared with T3, had weak binding ability to CXCR4 (0.6% and 9.9%) but almost equal binding ability to CCR5 (13.3% and 13.5%). C25 prefers to utilize R5 to bind cells. The activity enhancement caused by N425R mutation is still reflected on C25, but not on C27. The 194 associated mutant M22 (167-194V3-N425R) exhibits a twofold increase in overall activity over chimera C25 (167-194V3), which is absent in the 176 associated mutant M25 (167-176V3-N425R) and chimera C27 (167-176V3). The mutant M23 (167-194V3-S440D-I442R) obtained by introducing mutation S440D-I442R into C25 showed no positive signal both on Ghost cells and TZM-bl cells, showing loss of infective activity. Interestingly, mutation S440D-I442R, which did not confer elevated overall activity on C27 (176V3), but altered the infectious ratio of the two Ghost-CD4 cell lines, such that M26 (167-176V3-S440D-I442R) infected Ghost-CD4-CXCR4 much higher than the Ghost-CD4-CCR5 compared with C27.

## Discussion

In this study, we selected samples from HIV-1 infected patients CYM167 and CYM248, who respectively carried X4 and R5 viruses, to construct pseudoviruses. The obtained pseudoviruses from CYM167 and CYM248 are confirmed to be R5 and X4 tropic viral strains respectively. By mutually exchanging substations of their envelope gene regions, we constructed two panels of R5 to X4 forward switching and X4 to R5 reverse switching chimeric pseudoviruses. We determine the compensatory sites for coreceptor switching as well as the R5 to X4 evolutional pathway. V3 loop plays a critical role in the coreceptor switching and is also a sufficient condition for the shifting of tropism to occur. The substitution of the V3 region is accompanied by the decline of virus vitality. This phenomenon may suggest that the conversion of tropism from R5 to X4 needs to overcome a certain genetic barrier to make its virulence develop in a stronger direction at the cost of reducing the ability of infection. If the genetic barrier is successfully crossed, the virus will evolve into a more virulent virus with X4 tropism, resulting in a more rapid decline of CD4 and acceleration of the disease process. The most critical part for coreceptor-switching in HIV-1 is the V3 loop of gp120 [28], but it is inseparable from the assistance of other parts. In the previous study, we always think that V1V2 and V3 are associated with the virus tropism, but the chimeric pseudoviruses obtained by replacing any region of V1 to V3 in CRF01_AE subtype are inactive, which is contrary to the conclusion that substituting V1V2 region compensates for virus lethal mutation in previous studies of other HIV-1 subtypes [13,29]. The C4 region plays a very important role in improving pseudovirus fitness for coreceptor-switching [15]. Our results show that the presence of the C4 region is a sufficient condition for the pseudoviruses that shift in tropism and possess high replication activity. The role of the C4 region in the transition of tropism has been mentioned in other studies. The C4 region of gp120, as a part of the CD4-binding site, and its internal amino acid are mentioned that can synergistically interact with the V3 loop for tropism [30]. The stem of the V3 region is the most critical part for the utilization of coreceptors, while some residues in the V3 crown and C4 affect the interaction between envelope and coreceptor and membrane fusion [10,31]. This is also consistent with the preliminary conclusion of our experimental data.

Among the numerous mutants of C4, the S440D-I442R mutants conferred the enhancement of fitness in coreceptor-switching virus with V3 mutation, about twofold more active than other single or multiple point mutations. From the structural model, the 440–442 of C4 are the position spatially closest to coreceptors, and there may be some interactions between amino acids [32]. An interesting phenomenon is also observed in our results of Table 1 for positions 440–442 in the C4 region. Introducing this mutation into the dual-tropic pseudovirus p194, which without the S440D-I442R mutation, results in the attenuation of the ability to infect Ghost-CD4-CCR5 while leaving the ability to infect Ghost-CD4-CXCR4 unchanged. The introduction of this mutation in 167-176V3 could also render the pseudovirus less competent to infect Ghost-CD4-CCR5 and not less competent to Ghost-CD4-CXCR4. From this data, we can speculate that the existence of the S440D-I442R mutation can make the virus tend to utilize CXCR4 rather than CCR5. In other researchers’ analysis of the evolutionary orientation of amino acids in Env for 186 subtype B sequences, amino acid variation at residue 440 is linked with the usage of CXCR4 besides the V3 loop [33]. Another stronger evidence comes from the study demonstrated that a significant covariant association between negative charged amino acids at position 322 in V3 and positive charged at 440 in C4 contributes to the specificity of HIV-1 subtype B strains using CXCR4 [34]. The residue 442 has been confirmed by many studies as a coreceptor binding site outside the V3 loop [35,36]. As a part of the CD4-binding site, the 425 position in the C4 region, which contributes greatly to the overall replication activity after changing from an uncharged asparagine to a positively charged arginine, showed an eightfold elevation in the experimental results. When considering the nature of the coreceptor binding site, it may not be well exposed, and even not exist on HIV-1 env before CD4 binding. Hence substitutions that affect CD4 binding, or the ability of gp120 structural domains to undergo conformational changes, will also have an impact on the interactions of gp120 with both CCR5 and CXCR4 [2]. The exposure of neutralizing epitopes in the CD4-binding site may be another reason for more favourable coreceptor binding, which allows the V3 loop to be better exposed [37,38]. The above two characteristics also explain to some extent why alterations in the C4 region can increase the viral replication activity of the chimeric viruses.

Taken together, we started with an HIV-1 phenotypic study and used the biological phenomenon observed first to inversely explore the cause of the viral coreceptor switching in the CRF01_AE subtype. This differs from most previous studies that started with an analysis of viral genotypes to search for the critical site of the tropism. Based on previous studies by Song [23] on the CYM cohort, two pseudovirus clones with considerable vitality belonging to different evolutionary clusters of CRF01_AE and differing coreceptor utilization, p167 and p248, were derived. Based on these two clonal strains, multi-site, multi-fragment substitutions, and mutations were carried out to find the rule of the HIV-1 cell tropic transition. While supported by unambiguous data on coreceptor switching compared to the research starting with sequence analysis and prediction, there are still limitations in this study. Whether the data obtained from the alterations performed in the two basal clones selected for this study are population representative. Even though we later added the experimental data of two samples (CYM194 and CYM176) in the same cluster as CYM248, the results obtained were confirmatory of partial results rather than full concordance. Due to the limited samples, whether the conclusions and courses obtained in this study can be extended to other viruses in cluster 4 or 5 and the whole CRF01_AE subtype remains to be proved, which is also a direction of our future work. Another limitation is the difference between the structural simulation results and the actual integration model. All of our structural analyses are based on the cryo-electron microscopy structure 6meo, a structural basis of CCR5 recognition by HIV-1 envelope spike. The accuracy of this model in predicting the recognition pattern of another coreceptor, CXCR4, remains to be discussed.

In summary, we demonstrated that the minimal determinant for coreceptor switching includes two interacting domains: the V3 loop and the C4 domain of HIV-1 envelope protein. It has been reported in the past that the V3 loop is needed for binding to the proper coreceptor, but not much was known before for the C4 region’s compensate function for the Loss-of-Fitness caused by V3 loop mutations that happened during tropism switching. The mutagenesis of N425R was able to compensate for deleterious V3 mutation in viral vitality and S440-I442 mutant to the charged amino acids rendering it more competent for X4 tropism. This will be extremely advantageous for subsequent exploration of V3 region variants that are prone to loss of replication activity but critical for coreceptor switching. Understanding the changing pattern of CRF01_AE HIV-1 tropism transition might provide some reference for studying virus evolution and tropism switching for other HIV-1 subtypes and circulating recombinant forms. This study could also help building surveillance tools to collect HIV-1 phenotypic information and monitor tropism switching. Such information has strong public health impacts on improving HIV-1 antiviral therapy, since it could facilitate to selection of the right entry inhibitors, CCR5 or CXCR4 antagonists, to the right patients at the right time.
